# Mitigating combined toxic effects of arsenic, ammonia, and high temperature through dietary Iron in fish

**DOI:** 10.3389/fimmu.2026.1733912

**Published:** 2026-03-11

**Authors:** Neeraj Kumar, Paritosh Kumar, Kotha Sammi Reddy

**Affiliations:** ICAR-National Institute of Abiotic Stress Management, Baramati, India

**Keywords:** CYP 450, iNOS, iron, stress, TNFα, toxicity

## Abstract

**Background:**

The sustainability of aquaculture is increasingly threatened by major challenges such as aquatic pollution, excessive water abstraction, and climate change. Fish reared under such compromised environmental conditions often accumulate various contaminants, posing risks to consumer health. This study addresses these issues by formulating iron (Fe) based diets in *Pangasianodon hypophthalmus* reared under controlled conditions and simultaneously exposed to low levels of ammonia, arsenic, and high-temperature stress (NH_3_+As+T). However, the present investigation specifically focuses on the use of iron to mitigate the combined effects of ammonia, arsenic, and elevated temperature stress in *P. hypophthalmus*.

**Methods:**

An experiment was conducted to evaluate the efficacy of dietary Fe at 40, 50, and 60 mg kg^-^¹ in mitigating the concurrent toxicity of ammonia, arsenic, and high temperature in P. hypophthalmus. A total of 360 fish were used in this study. Each treatment included 45 fish, with 15 fish stocked per replicate. Total RNA was isolated and quantified using the TRIzol method, followed by cDNA synthesis and quantitative PCR to assess differential gene regulation. Physiological parameters, protein and carbohydrate metabolic enzymes, cortisol levels, and immunological markers were analyzed. Additionally, arsenic bioaccumulation and DNA damage (single-cell gel electrophoresis) were evaluated.

**Results:**

The genes *HSP70*, *CYP450*, *Caspase 3a* and *3b*, *iNOS*, *MT*, and *Na^+^/K^+^-ATPase* in liver tissue were markedly upregulated in fish exposed to the combined stressors (NH_3_+As+T). Notably, these genes were also significantly upregulated in the group supplemented with 50 mg kg^-^¹ Fe compared to control and stressed groups. Furthermore, immune-related genes such as *TNF-α, IL, Ig*, and *TLR* showed improvement with Fe supplementation. In contrast, growth-related genes including *GH, GHR1, GHRβ, IGF1X, IGF2X, SMT, and MYST* were significantly altered by exposure to the stressors.

**Conclusions:**

Overall, the findings demonstrate the potential of dietary iron as an effective strategy to enhance fish health and physiological resilience under multiple environmental stressors. The study provides mechanistic insights into how Fe supplementation modulates gene expression and cellular metabolic pathways to mitigate the toxic effects of ammonia, arsenic, and high temperature in *Pangasianodon hypophthalmus*.

## 1 Introduction

Aquatic ecosystems are profoundly influenced by a combination of abiotic and biotic factors, which collectively determine the productivity and health of aquatic organisms. Since from last few years, the concentrations of inorganic pollutants and various waterborne contaminants have risen sharply, negatively impacting fish performance and quality (1). Among these stressors, temperature fluctuations and the presence of toxic pollutants stand out as critical abiotic factors contributing to the decline in food production across aquatic ecosystems. (In aquaculture system the acceptable levels of total ammonium nitrogen (TAN) are often 0.5 to 2.0 mg L^-1^). The unionized ammonia that induces toxicity in fish (2). High concentrations of ammonia are particularly harmful to fish, leading to reduced growth performance, suppressed immunity, tissue deformities, and mass mortality (3, 4). Due to ammonia toxicity, it induces oxidative stress, neurotoxicity, and impaired oxygen delivery (5–7). Among abiotic factors, temperature is especially crucial, as fish are poikilothermic animals. Temperature not only affects fish directly but also influences the toxicity of ammonia. The higher temperatures increase the bioavailability, diffusion rate, and chemical reactivity of ammonia in fish, thereby exacerbating its toxic effects (8, 9).

Arsenic (As) is one of the most dangerous metalloids found in aquatic ecosystems, posing serious health risks to fish, animals, and humans. The origin and distribution of arsenic are primarily due to anthropogenic and geogenic activities, such as pesticide application, burning fossil fuels, and mining (10). Arsenic concentrations have been documented to reach levels as high as 800 to 2500 ppm in several countries, including Bangladesh and Chile (11). Arsenic occurs in both organic and inorganic forms and can be further categorized based on its oxidation states, including arsenate (As^5^^+^), arsenite (As³^+^), arsine (AsH_3_) and elemental arsenic (As^0^) (12). Additionally, arsenic has methylated derivatives known as “fish arsenic” (arsenocholine-AsC and arsenobetaine-AsB) and arsenosugar compounds (12). Nearly 200 million people face significant health risks due to arsenic contamination, resulting in an estimated 43,000 deaths each year in Bangladesh (13, 14). The International Agency for Research on Cancer (IARC) classifies arsenic as a Group 1 carcinogen, indicating its confirmed potential to cause cancer in humans (15). The combined effects of ammonia, high-temperature stress, and arsenic pollution create severe conditions in aquatic ecosystems, as highlighted in the present study.

Interestingly, iron (Fe) has great potential to reduce the impact of stressors on aquatic organisms (16). In the current scenario of climate change and altered conditions in aquatic ecosystems, Fe could be a boon for aquatic animals in coping with these challenges. Iron is a vital micronutrient that plays a critical role in various biological functions in fish, including DNA synthesis, energy metabolism and oxygen transport (17–19). Iron is also integral to cellular metabolism, primarily through its incorporation into heme proteins that facilitate oxygen binding and drive the mitochondrial electron transport chain (20, 21). Iron in its ferrous form (Fe²^+^) plays a key role in transport mechanisms mediated by divalent metal transporter 1 (DMT1), also known as Nramp2, and by the iron exporter ferroportin, also referred to as iron-regulated transporter (IREG). These transporters are essential for the basolateral transport of iron (22–24). Fe-containing diets can improve gene regulation related to antioxidative status, immunity, growth performance, and cellular metabolic stress, as well as enhance the detoxification of heavy metals in fish reared under various abiotic and biotic factors.

Heat shock proteins (*HSPs*) are generally expressed during stress to help cells recover from damage (25). Similarly, cortisol, secreted by head kidney cells, is the primary stress response indicator in fish (26). Antioxidant enzymes encoded by genes such as superoxide dismutase (*SOD*), catalase (*CAT*), and glutathione peroxidase *(GPx*) play a vital role in mitigating oxidative stress by neutralizing excess free radicals generated under stressful conditions (27). Apoptosis and detoxification genes like *Caspase 3a (Cas 3a), Caspase 3b (Cas 3b)*, and *cytochrome P450 (CYP 450)* play important roles in controlling uncontrolled cell growth and enhancing the detoxification of contaminants within the fish body, respectively (28) (Elmore, 2007). Growth performance genes, including growth hormone receptor 1 (*GHR1*), growth hormone (*GH*), growth hormone receptor beta (*GHRβ*), somatostatin (*SMT*), and myostatin (*MYST*) are significantly affected by pollutants and other stress factors (29). Immunological genes, such as tumor necrosis factor (*TNFα*), interleukin (*IL*), total immunoglobulin (*Ig*), and toll-like receptor (*TLR*), are also notably impacted by abiotic and biotic factors (6).

*Pangasianodon hypophthalmus* is an economically important fish species with widespread cultivation around the globe. It is well-suited for evaluating the effects of various abiotic factors (29). This species is known for its high growth rate, adaptability, market demand, sturdiness, and medicinal value. It is highly suitable for intensive aquaculture, with global production reaching 2,520.4 thousand tons in 2020 (30).

The objectives of the study are:

1. To investigate the mitigating potential of dietary iron against concurrent exposure to low doses of ammonia, arsenic, and elevated temperature.
2. To evaluate the role of dietary iron in regulating the expression of genes associated with immune function, antioxidant defense, growth performance, genotoxicity, apoptosis, and detoxification, thereby mitigating multiple abiotic stressors in *Pangasianodon hypophthalmus*.

## 2 Material methods

### 2.1 Ethics statement

The fish experimentation facilities are registered with the Committee for the Purpose of Control and Supervision of Experiments on Animals (CCSEA) under registration number 2190/GO/RReBi/SL/2022. The study methodology and protocol strictly adhered to the Animal Research: Reporting of *In Vivo* Experiments (ARRIVE) guidelines.

### 2.2 Experimental animal and design

Healthy *Pangasianodon hypophthalmus* (average weight: 6.02 ± 0.24 g; average length: 5.12 ± 0.18 cm) were obtained from the ICAR-NIASM fish pond. Prior to the experiment, the fish were quarantined using potassium permanganate (KMnO_4_) and 1% salt dip solution. The study was conducted using a completely randomized design (CRD) comprising eight treatment groups, each in triplicate. Three hundred sixty fish were used in this experiment. Each treatment consisted of forty-five fish, with fifteen fish maintained in each replicate. The capacity of tank was 150 L for commencement of the experiment. The details of the experimental designs has been mentioned in the **Table 1**. The experimental treatments were categorized as follows: 

**Table 1:** Details of experimental design.

S. no	Details of the treatments	Notation
1	Control	Ctr
2	Concurrent exposure to arsenic, ammonia and high temperature and fed with control diet	As+NH_3_+T
3	Fed with Iron at 40 mg kg^-1^ diet	Fe at 40 mg kg^-1^ diet
4	Fed with Iron at 50 mg kg^-1^ diet	Fe at 50 mg kg^-1^ diet
5	Fed with Iron at 60 mg kg^-1^ diet	Fe at 60 mg kg^-1^ diet
6	Concurrent exposure to arsenic, ammonia and high temperature and fed with Iron at 40 mg kg^-1^ diet	Fe at 40 mg kg^-1^ diet+As+NH_3_+T
7	Concurrent exposure to arsenic, ammonia and high temperature and fed with Iron at 50 mg kg^-1^ diet	Fe at 50 mg kg^-1^ diet+As+NH_3_+T
8	Concurrent exposure to arsenic, ammonia and high temperature and fed with Iron at 60 mg kg^-1^ diet	Fe at 60 mg kg^-1^ diet+As+NH_3_+T

The experimental diets (Iron) fed twice a day at 9:00 AM and 5:00 PM. The fecal maters and uneaten feed were removed by siphoning on daily basis. The experimental water quality parameters were checked on a regular basis (31), which is suitable for culture of *P. hypophthalmus* (29). The experimental water (2/3^rd^ parts) was exchanged every alternate days and maintained the concentration of sodium arsenite (NaAsO_2_) (96 h, LC_50_ 26.88 mg L^-1^; 1/10^th^ of LC_50_:2.68 mg L^-1^) and (NH_4_)_2_SO_4_ (96 h, LC_50_ 20.0 mg L^-1^; 1/10^th^ of LC_50_: 2.0 mg L^-1^) (5, 32). The high temperature (34 ± 0.25 °C) was maintained by thermostatic heater. The stock solution for arsenic and (NH_4_)_2_SO_4_ was 100 mg L^-1^. The details for determination of LC_50_ and stock preparation of both the chemical were reported in our previous finding. Four iron containing iso-nitrogenous (35% crude protein) and iso-caloric experimental diets were prepared such as Fe 0, 40, 50 and 60 mg kg^-1^ diet. The local feed ingredients used for feed preparation and mentioned (33) in **Table 2**. The gross energy was calculated using Halver (34) method of the diets.

**Table 2:** Ingredients composition and proximate analysis of experimental diets (% dry matter) of iron (Fe), fed to *Pangasianodon hypophthalmus* for 90 days.

Feed ingredients	Fe-0 mg kg^-1^ diet	Fe-40 mg kg^-1^ diet	Fe-50 mg kg^-1^ diet	Fe-60 mg kg^-1^ diet
Soybean meal^a^	35.5	35.5	35.5	35.5
Fish meal^a^	25	25	25	25
Groundnut meal^a^	10	10	10	10
Wheat flour^a^	17.47	17.466	17.465	17.464
Sunflower oil^a^	4.5	4.5	4.5	4.5
Cod liver oil^a^	1.5	1.5	1.5	1.5
CMC^b^	2	2	2	2
Vitamin and mineral mix*	2	2	2	2
Vitamin C^c^	0.03	0.03	0.03	0.03
Lecithin^b^	2	2	2	2
Fe	0	0.004	0.005	0.006
Proximate composition of the diets				
Crude protein (CP)	34.92 ± 0.10	35.13 ± 0.02	35.14 ± 0.09	34.95 ± 0.33
Ether extract (EE)	9.16 ± 0.03	9.27 ± 0.08	9.49 ± 0.11	9.34 ± 0.06
Total carbohydrate (TC)	38.28 ± 0.13	38.17 ± 0.45	36.69 ± 0.37	37.60 ± 0.12
Organic matter (OM)	90.43 ± 0.06	90.52 ± 0.20	90.40 ± 0.13	90.33 ± 0.20
Dry matter (DM)	91.34 ± 0.02	91.30 ± 0.04	90.81 ± 0.50	90.43 ± 0.17
Digestible energy (DE)	356.96 ± 0.32	358.11 ± 2.06	353.72 ± 1.35	355.58 ± 1.41
Iron (Fe, mg kg^-1^)	2.62 ± 0.13	45.22 ± 0.18	56.38 ± 0.20	63.98 ± 0.36

a Procured from local market,

b Himedia Ltd, Himedia Ltd,

c SD Fine Chemicals Ltd., India.

*Manual prepared Vitamin mineral mixture; Composition of vitamin mineral mix (quantity/250 g starch powder): vitamin A 55,00,00 IU; vitamin D3 11,00,00 IU; vitamin B1:20 mg; vitamin E 75 mg; vitamin K 1,00 mg; vitamin B12 0.6 mcg; calcium pantothenate 2,50 mg; nicotinamide 1000 mg; pyridoxine: 100 mg; Zn 500 mg; I 1,00 mg; Mn: 100 mg, Cu 200 mg; Co 45 mg; Ca 50 g; P 30 g; Se: 2 ppm.

Digestible energy (DE) (Kcal/100 g) = (% CP × 4) + (% EE × 9) + (TC × 4).

Data expressed as mean ± SE, n = 3.

Total carbohydrate % =100- (Ether extract (EE %)+Crude protein (CP %)+ Ash (%)+Moisture).

### 2.3 RNA isolation and quantification

Fish were anesthetized with clove oil (100 µl L^-1^) before collecting the liver tissue of *P. hypophthalmus* for RNA isolation using the TRIzol reagent protocol (Catalogue no. 15596018; Invitrogen™, Life Technologies Corporation, Carlsbad, California 92008, USA). Initially, the tissue was snap-frozen and ground in liquid nitrogen to ensure thorough homogenization. Chloroform was added to the homogenate to facilitate phase separation, followed by a brief incubation period of 5 minutes. Subsequent centrifugation ((Eppendorf AG, 5430R, Ham burg, Germany) allowed for the collection of the RNA rich aqueous phase, which was carefully transferred to a sterile 1.5 mL microcentrifuge tube. RNA was then precipitated with isopropanol, air-dried, and rehydrated in RNase-free water. To enhance purity, the RNA was washed with 75% ethanol before being stored at -80 °C until further use. RNA integrity was verified via 1.0% agarose gel electrophoresis using 1× TAE buffer, with band visualization conducted through the ChemiDoc™ MP Imaging System (ChemiDocTM MP imaging system, Bio-Rad). Quantification and purity assessment of the RNA samples were carried out using a NanoDrop spectrophotometer (Thermo Scientific, NanoDrop Lite Plus Spectrophotometer, Serial No. NLP2200122).

### 2.4 cDNA synthesis and quantitative PCR

Complementary DNA (cDNA) was synthesized from the extracted total RNA using the RevertAid First Strand cDNA Synthesis Kit (Catalog number, K1622, Thermo Fisher Scientific Baltics UAB, Lithuania, Europe). Prior to synthesis, residual genomic DNA was removed using DNase I treatment. The initial reaction mixture (12 µL) contained 100 ng of RNA and 15 pmol of oligo(dT) primers. This mixture was heated at 65 °C for 5 minutes and immediately chilled on ice. After a brief centrifugation step, the reverse transcription reaction was assembled by adding 1 µL of RiboLock RNase Inhibitor (20 U/µL), 1 µL of RevertAid Reverse Transcriptase, 4 µL of 5× reaction buffer, and 2 µL of 10 mM dNTP mix to the RNA template. The reaction mixture was incubated at 60 °C for 42 minutes to facilitate cDNA synthesis, followed by enzyme inactivation at 70 °C for 5 minutes. The quality of the synthesized cDNA was validated through PCR (BioRAD: Model C1000 Touch Thermo Cycler, Serial No. CT067159, RFP45131) amplification of the housekeeping gene *β-actin*. Quantitative real-time PCR (qPCR) (Quant Studios 5, Applied Biosystems. Model A 28134; Serial No. 2725212054; Thermo Fisher Scientific San Francisco) was then carried out using gene-specific primers and SYBR Green PCR Master Mix (Bio-Rad). Each qPCR reaction comprised 1× SYBR Green Master Mix, 1 µL each of forward and reverse primers, and 1 µL of cDNA template. Thermal cycling conditions included an initial denaturation at 95 °C for 10 minutes, followed by 39 cycles of amplification with denaturation at 95 °C for 15 seconds and annealing/extension at 60 °C for 1 minute (35). The primer sequences used for gene expression analysis are provided in **Table 3**.

**Table 3:** Details of primer for relative quantitative real-time PCR.

Gene	Primer sequence (5′ –3′)	Accession number	PCR Efficiency
*SOD*	F-GTCCATCTTACCCGGTGCCC R-CGAGAGAAGACCCGGAACGC	XM_034299545.1	1.41
*CAT*	F-AGCAGGCGGAGAAGTACCCA R-GCTGCTCCACCTCAGCGAAA	XM_026919141.2	1.40
*GPx*	F- GTCACTGCAGGATGCAACAC R- TTGGAATTCCGCTCATTGAT	XM_026947312.2	1.45
*HSP 70*	F- CTCCTCCTAAACCCCGAGTC R- CCACCAGCACGTTAAACACA	XM_026934573.2	1.54
*iNOS*	F-ACACCACGGAGTGTGTTCGT R-GGATGCATGGGACGTTGCTG	XM_026931613.2	1.48
*DNA damage inducible protein*	F-CACCTTCGCCCTCGAAGTCT R-GCTCGGGTGAGGTCTCTCAG	XM_026938137.2	1.39
*TNFα*	F-TGGAGTTCTGCTTGCCGTGG R-GCAGCCTTTGCAGTCTCGGA	XM_026942329.2	1.40
*TLR*	F: TCACCACGAACGAGACTTCATCC R: GACAGCACGAAGACACAGCATC	XM_026916808.2	1.45
*Ghr1*	FTATTGGCTACAGCTCGCCGC R-AATCACCCCGACTGTGCTGC	XM_034306157.1	1.46
*Ghrb*	F-TTGAGCTTTGGGACTCGGAC R-CGTCGATCTTCTCGGTGAGG	XM_026942987.2	1.68
*IGF-1X1*	F-GCAACGGCACACAGACACGC R-CAGACGTTCCCTCACCATCCTCT	XM_034313382.2	1.50
*IGF-1X2*	F-CGAGAGCAACGGCACACAGA R-TTCTGATGGACCTCCTTACAAGATG	XM_034313383.2	1.44
*IL*	F- AGCAGGATCCATCAAAGTGG R- GTGCTCCAGCTCTCTGGGTA	XM_026918084.2	1.49
*Ig*	F- GGCCAGTAATCGTACCTCCA R- CTTCGTAAGGTCCCCACTGA	XM_026923540.2	1.57
*MYST*	F-GGGAAAGACCTGGCCGTGAC R-TCGAGGCCGGATTCTCGTCT	XM_026910492.2	1.56
*SMT*	F- CTCTGGGTGGCAGAATGAAT R- AACATGAAGAGAACGTTTTCCAG	XM_026921272.2	1.72
*GH*	F-CCCAGCAAGAACCTCGGCAA R-GCGGAGCCAGAGAGTCGTTC	GQ859589.1	1.48
*CYP P450*	F-GATTCGGCATCCGTGCGTGC R-GATGTGGCTGGGACGAGCA	NC_047599.1	1.50
*MT*	F-CACGGCTTTTCCTGTCCGCT R-AACAGCGCCCCCAGGTGTC	AF087935.1	1.57
*Cas 3a*	F-CGGCATGAACCAGCGCAAC R-TCCACCGCACCATCTGTCCC	NC_047622.1	1.42
*Cas3b*	F-AGCTTTCCGTGAGCTGGGCT R-TGGCTGACTTGCTGTGGTCCT	NC_047601.1	1.44
*Na^+^K^+^ATPase*	F-AACTACAAGCCCACGTACCA R-CTTGCCAGCCTTAAAGCCAA	XM_026923907.3	1.42
*β-Actin*	F-CAGCAAGCAGGAGTACGATG R-TGTGTGGTGTGTGGTTGTTTTG	XM_031749543.1	

*SOD*, Superoxide dismutase; *CAT*, Catalase; *GPx*, Glutathione peroxidase; *HSP*, Heat shock protein; *iNOS*, Nitric oxide synthase; *TNFα*, Tumor necrosis factor; *TLR*, Toll like receptor; *Ghr*, Growth hormone receptor; *IL*, Interleukin; Ig, Immunoglobulin; *MYST*, myostatin *SMT*, Somatostatin; *CYP P450*, Cytochrome P450; *MT*, Metallothionine; *Cas 3a* and *3b*, caspase 3; *GH*, Growth hormone; *IGF1* and 2, Insulin like growth factor.

### 2.5 Growth performance

Growth performance parameters assessed in this study included feed conversion ratio (FCR), percent weight gain, specific growth rate (SGR), protein efficiency ratio (PER), relative feed intake (RFI), thermal growth coefficient (TGC), and daily growth index. Fish were weighed at 15-day intervals throughout the 90-day experimental period to monitor and evaluate these growth metrics.

SGR = 100 (ln FBW-ln IBW)/number of days

FCR = Total dry feed intake (g)/Wet weight gain (g)

Weight gain (%) = Final body weight (FBW)-Initial body weight (IBW)/Initial body weight (IBW) ×100

Relative feed intake, (FI) (%/d) = 100 × (TFI/ιBW)

PER= Total wet weight gain (g)/crude protein intake (g)

Daily growth index, DGI (%) = (FBW^1/3^ – IBW^1/3^)/days × 100

### 2.6 Neurotransmitter enzyme activities

Acetylcholinesterase (AChE) activity (EC 3.1.1.7) was quantified in brain using a modified protocol based on the method developed by Augustinsson and later adapted by Hestrin (36). The reaction mixture included 1 mL of phosphate buffer, 1 mL of acetylcholine solution, and 0.2 mL of the sample extract. This mixture was incubated at 37 °C for 30 minutes to allow enzymatic activity. To terminate the reaction and facilitate color development, alkaline hydroxylamine, hydrochloric acid (HCl), and ferric chloride were sequentially added. The resulting absorbance was measured at 540 nm to determine AChE activity.

### 2.7 Cortisol

Cortisol levels were determined using a commercially available ELISA kit (Cortisol EIA Kit, Cat. No. 500360; Cayman Chemicals, USA), following the manufacturer’s protocol. Absorbance values were recorded using an ELISA plate reader (Biotek, India Pvt. Ltd.) to obtain the final reading.

### 2.8 Respiratory burst activity, serum protein, A:G ratio and blood glucose

Respiratory burst activity was evaluated using the method originally described by Secombes and later modified by Stasiack and Baumann (37). Total plasma protein concentration was measured colorimetrically using the BCA method with a commercial protein assay kit (Erba Total Protein Kit, Code no. 120231). Albumin levels were determined based on the bromocresol green binding technique as outlined by Doumas et al. (38). Globulin concentration was calculated by subtracting the albumin value from the total plasma protein, and the albumin-to-globulin (A/G) ratio was derived by dividing albumin by globulin. Blood glucose levels were estimated following the protocols of Nelson and Somogyi (39). In this method, blood samples were deproteinized using zinc sulfate and barium hydroxide, followed by filtration. The glucose concentration in the resulting supernatant was measured spectrophotometrically at 540 nm using a reagent blank as reference.

### 2.9 Myeloperoxidase content

Serum myeloperoxidase (MPO) activity was measured according to Quade and Roth (40) with minor modifications. Briefly, 30 μL of serum was diluted with 370 μL of Ca²^+^- and Mg²^+^-free Hank’s Balanced Salt Solution (HBSS) in Eppendorf tubes. Then, 100 μL of 0.1 mg mL^-^¹ 3,3′,5,5′-tetramethylbenzidine dihydrochloride and 0.006% freshly prepared hydrogen peroxide were added. The reaction was monitored kinetically at 50-s intervals for 4.5 min. Reaction velocity was expressed as international units (IU), defined as the amount of enzyme required to produce an increase in absorbance of 0.001 per minute in 0.5 mL of reaction mixture (ΔA_450_ min^-^¹ mL^-^¹).

### 2.10 Aspartate aminotransferase and alanine aminotransferase, lactate dehydrogenase, and malate dehydrogenase

The activities of aspartate aminotransferase (AST; EC 2.6.1.1) and alanine aminotransferase (ALT; EC 2.6.1.2) were measured following the protocol described by Wootton (41). Stock solutions of potassium dihydrogen phosphate, dipotassium hydrogen phosphate, 1N sodium hydroxide, and 2,4-dinitrophenylhydrazine (DNPH) were prepared in advance. For the ALT assay, α-ketoglutarate and DL-alanine served as substrates, whereas the AST assay utilized α-ketoglutarate and DL-aspartic acid. The substrate solution was combined with tissue homogenate and incubated at 37 °C for one hour. Following incubation, DNPH was added to the mixture, and absorbance was measured at 540 nm. Subsequently, 0.4 N NaOH was introduced to complete the reaction. Lactate dehydrogenase (LDH) activity was determined using the method of Wroblewski and Ladue (42). The reaction mixture consisted of 0.1 M phosphate buffer prepared from sodium dihydrogen phosphate and disodium hydrogen phosphate along with freshly prepared NADH and sodium pyruvate as substrates. After adding the enzyme extract, the mixture was incubated for 20 minutes before measuring absorbance at 320 nm. Malate dehydrogenase (MDH) activity was assayed according to Ochoa (43). The reaction conditions were similar to those used for LDH, with the exception that oxaloacetate was substituted for sodium pyruvate as the substrate.

### 2.11 Arsenic and iron analysis from fish tissues, feed and experimental water

Arsenic concentrations were determined in experimental water as well as in different fish tissues, including liver, muscle, gill, brain, and kidney, across all treatment groups. Additionally, iron (Fe) content was measured in the fish feeds. Water samples were first filtered through 0.45 µm membrane filters and then acidified by adding 100 µL of concentrated nitric acid (HNO_3_, 69%; Himedia Laboratory Pvt. Ltd., Mumbai, India). Fish tissues and feed samples underwent microwave-assisted digestion using a Multiwave PRO system (Microwave Reaction System, Multiwave PRO, Anton Paar GmbH, Austria, Europe), employing a digestion mixture of nitric acid and hydrogen peroxide in a 5:1 ratio. Once digestion was complete, samples were cooled to room temperature, filtered through Whatman filter paper (0.45 µm pore size), and diluted to a final volume of 50 mL. Quantification of arsenic in tissues and iron in feeds was performed by inductively coupled plasma mass spectrometry (ICP-MS; Agilent 7700 series, Agilent Technologies, USA), following the methodology outlined by Kumar et al. (44, 45).

### 2.12 Alkaline single-cell gel electrophoresis/comet assay

DNA damage in kidney tissue was evaluated using the alkaline single-cell gel electrophoresis (comet) assay, based on the method by Ali et al. (46) with slight modifications as described by Kumar et al. (27). Approximately 50 mg of kidney tissue was rinsed twice with double-distilled water, followed by two washes with chilled, calcium- and magnesium-free phosphate-buffered saline (PBS). The tissue was then transferred to an ice-cold homogenization buffer composed of 20 mM EDTA, 1× Hanks’ balanced salt solution, and 10% dimethyl sulfoxide (DMSO), adjusted to pH 7.0–7.5. A single-cell suspension was prepared and centrifuged at 3000 rpm for 5 minutes at 4 °C to pellet the cells, which were subsequently resuspended in PBS. Cell viability was assessed using the trypan blue exclusion assay (Ali et al., 2008). To prepare comet assay slides, 200 µL of 1% normal agarose was first layered onto a glass slide. This was followed by the addition of approximately 20,000 cells suspended in 85 µL of 0.5% low melting point agarose. A coverslip was placed on top briefly and then removed, after which the slide was overlaid with an additional 100 µL of 0.5% low melting point agarose. Slides were immersed overnight at 4 °C in a lysis solution containing 100 mM Na_2_EDTA, 2.5 M NaCl, 10 mM Tris (pH 10.0), supplemented with 10% DMSO and 1% freshly added Triton X-100. Following lysis, slides were placed in a horizontal electrophoresis chamber filled with alkaline buffer (1 mM Na_2_EDTA, 300 mM NaOH, 0.2% DMSO; pH>13.5) and subjected to electrophoresis at 15 V (0.8 V/cm) and 300 mA for 20 minutes at 4 °C. After electrophoresis, slides were neutralized by washing three times with 0.4 M Tris buffer (pH 7.5). DNA was then stained with 75 µL of ethidium bromide (20 µg/mL) for 5 minutes. The extent of DNA damage was visualized under a fluorescence microscope (Leica Microsystems Ltd, DM2000, Heerbrugg, Switzerland), with images captured and analyzed using OpenComet software. DNA damage was quantified as the percentage of tail DNA, calculated as % tail DNA = 100% − % head DNA.

### 2.13 Statistics

Data were analyzed using Statistical Package for Social Sciences program version 16.0 (SPSS Inc., Chicago, IL, USA). The data were expressed as mean ± standard error of mean and tested for normality and homogeneity of variance using the Shapiro-Wilk’s and Levene’s test, respectively. When both tests were satisfied, one-way ANOVA (Analysis of variance) with Duncan’s multiple range tests (DMRT) was employed to test the statistically significant difference at p<0.05.

## 3 Results

### 3.1 Cortisol, heat shock protein and *DDIP* affected by concurrent exposure to low dose of ammonia, arsenic and high temperature stress and iron diet protect it

**Figure 1:**
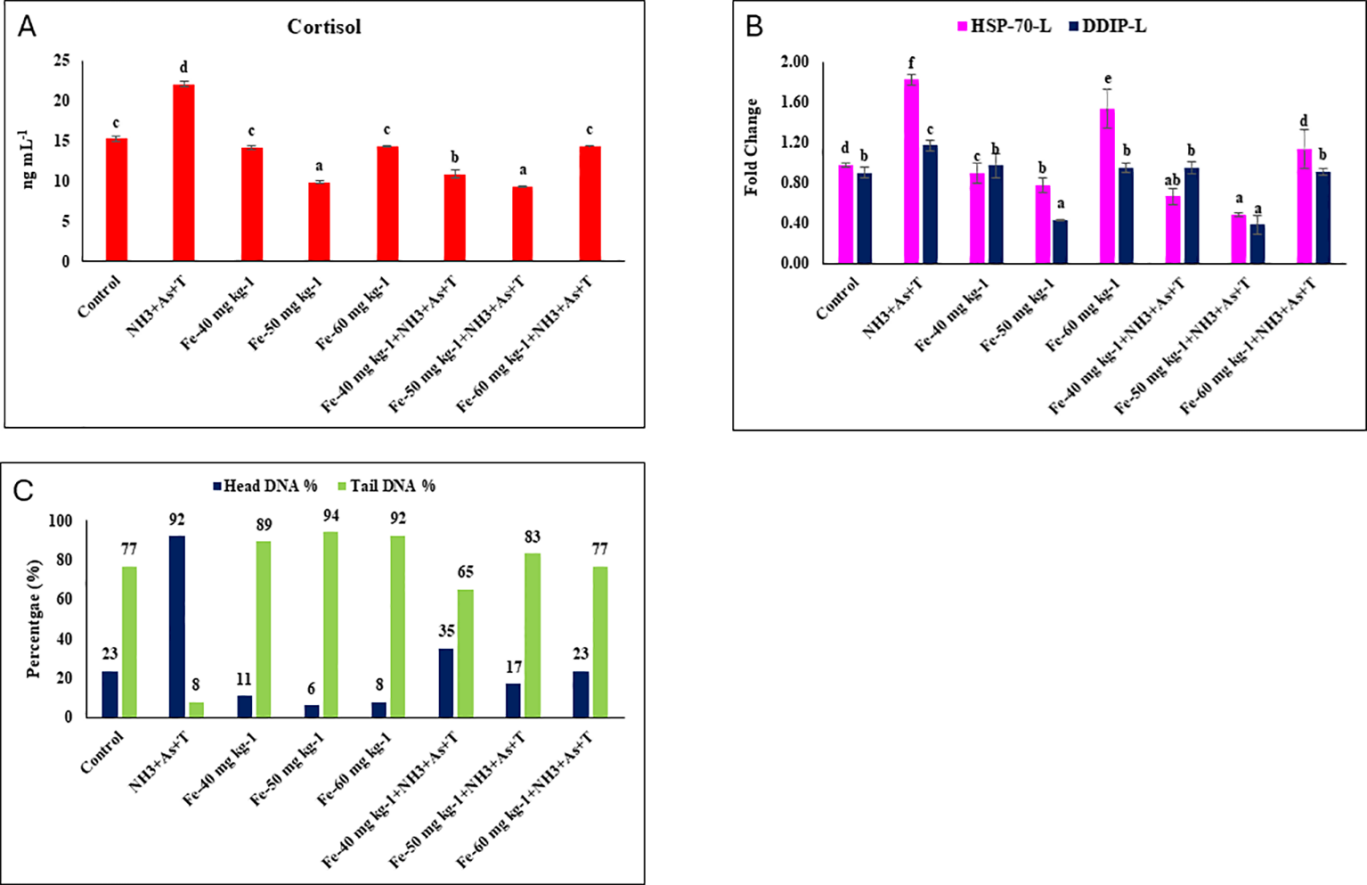
**(A–C)** Effect of dietary iron on cortisol, gene expression of HSP. 65, 70, DNA damage inducible protein (DDIP) in liver and comet assay (Head 60 % DNA and Tail % DNA) in fish reread under control or multiple stressors. Within endpoints and groups, bars with different superscripts di ffer si gnificantly (a-f). Data expressed as Mean ± SE (n=3).

A diet supplemented with iron (Fe) at 50 mg kg^-^¹, with or without stressors, significantly reduced cortisol levels (p = 0.0014) and provide protection against low doses of ammonia, arsenic, and high-temperature stress, comparable to the control and other treatment groups (**Figure 1A**). In contrast, diets containing Fe at 40 and 60 mg kg^-^¹ were less effective in regulating cortisol levels. Further, *HSP 70* gene expression in liver tissue was significantly downregulated (p = 0.0011) in fish fed with 50 mg kg^-^¹ diet, irrespective of stressor exposure (NH_3_+As+T), compared to the control and other groups. Conversely, fish exposed to the stressors exhibited a marked upregulation of *HSP70* expression compared control and Fe supplemented groups (**Figure 1B**). Similarly, *DDIP* gene expression was significantly downregulated (p = 0.011) with Fe at 50 mg kg^-^¹ diet, with or without stressors, compared to control and other groups. The groups fed with Fe at 40 mg kg^-1^ and 60 mg kg^-1^ had similar *DDIP* gene expression levels. However, *DDIP* expression was substantially upregulated in the stressor-exposed group (**Figure 1B**). Furthermore, Fe supplemented diets mitigated DNA damage, as evidenced by reduced tail and increased head DNA percentages. DNA damage was lowest in the group fed with Fe at 50 mg kg^-^¹ (6%), followed by the Fe at 60 mg kg^-^¹ (8%) and 40 mg kg^-^¹ (11%) groups. Under stressor exposure, DNA damage was reduced to 35%, 17%, and 23% in fish fed with Fe at 40, 50, and 60 mg kg^-^¹, respectively (**Figure 1C**).

### 3.2 *CYP 450, iNOS, Caspase* and metallothionein and *Na^+^K^+^ATPase* affected by concurrent exposure to low dose of ammonia, arsenic and high temperature stress and Iron diet protect it

**Figure 2:**
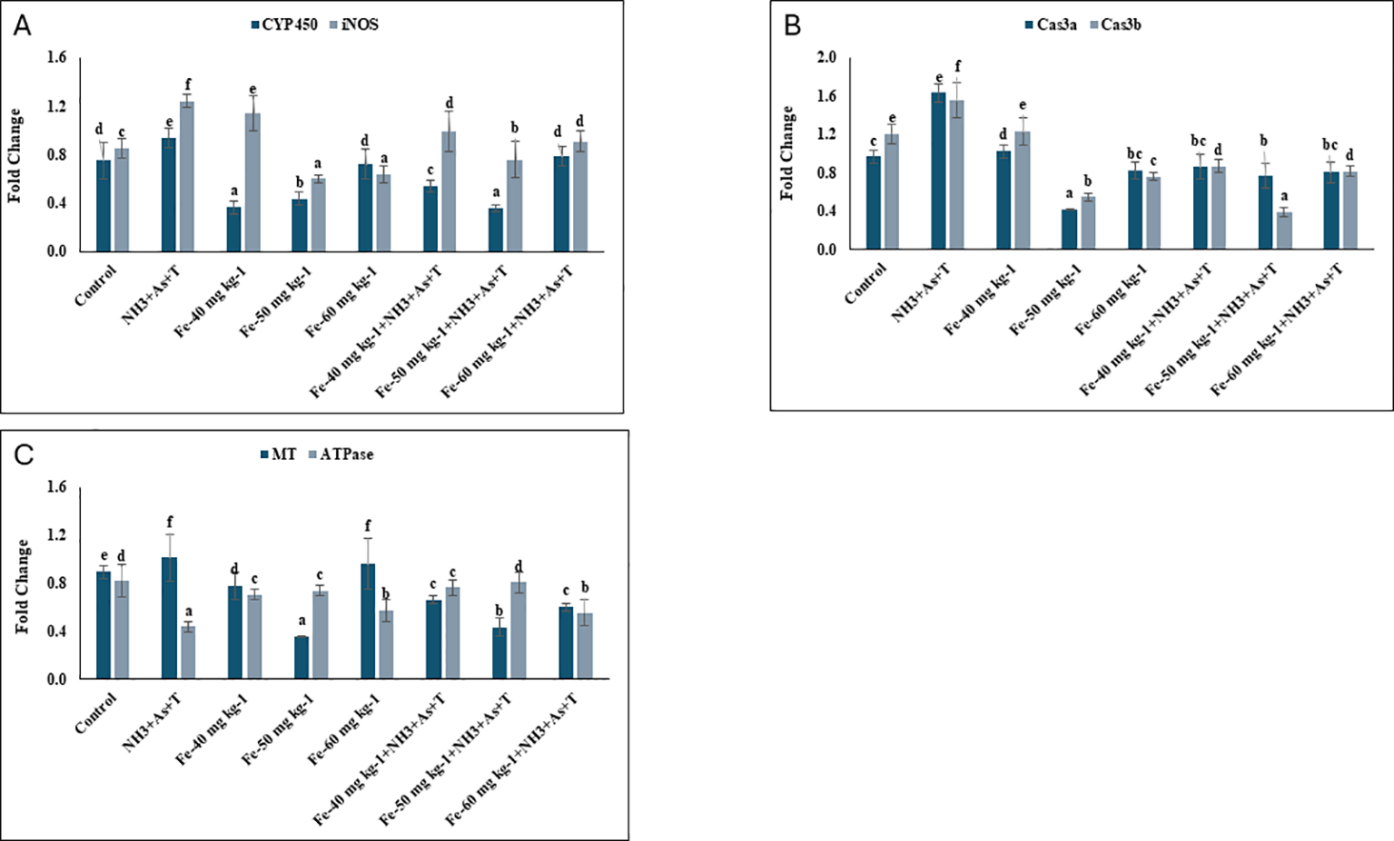
**(A–C)** Effect of dietary iron on gene expression of CYP 450, iNOS, Caspase (Cas 3a and 3b), MT and Na+K+ATPase in fish reread (under control or multiple stressors. Within endpoints and groups, bars with different superscripts differ significantly (a-f). Data expressed as Mean ± T SE (n=3).

*CYP450* gene in liver tissue was significantly downregulated (p = 0.001) in Fe supplemented diets at 40 mg kg^-^¹ and 50 mg kg^-^¹ (without stressors), as well as in those fed 50 mg kg^-^¹ Fe with stressors, compared to the control and other groups. In contrast, *CYP450* expression was markedly upregulated in the group exposed to combined stressors (NH_3_+As+T) (**Figure 2A**). Similarly, *iNOS* expression was significantly downregulated (p= 0.001) in fish fed with Fe at 50 mg kg^-^¹ and 60 mg kg^-^¹ without stressors, followed by those fed 50 mg kg^-^¹ Fe with stressors. *iNOS* upregulation occurred in the stressor-exposed group compared to control and Fe fed groups (**Figure 2A**). *Caspase 3a* (*Cas 3a)* was significantly downregulated (p=0.0016) in fish fed Fe at 50 mg kg^-^¹, with or without stressors, compared to the control and other groups. In contrast, *Caspase 3b* (*Cas 3b*) expression was significantly downregulated (p=0.0022) across all Fe-fed groups (40, 50, and 60 mg kg^-^¹), regardless of stressor exposure, relative to the control and stressor groups only. Both *Cas 3a* and *Cas 3b* were significantly upregulated in fish exposed to stressors (**Figure 2B**). Furthermore, the expression of metallothionein (*MT*) (p = 0.0016) and *Na^+^/K^+^-ATPase* (p = 0.018) genes was significantly upregulated in the stressor group compared to the control and Fe supplemented groups. However, supplementation with Fe at 50 mg kg^-^¹, with or without stressors, led to the downregulation of both *MT* and *Na^+^/K^+^-ATPase* expression (**Figure 2C**).

### 3.3 *TNFα, IL, Ig* and *TLR* affected by concurrent exposure to low dose of ammonia, arsenic and high temperature stress and iron diet protect it

**Figure 3:**
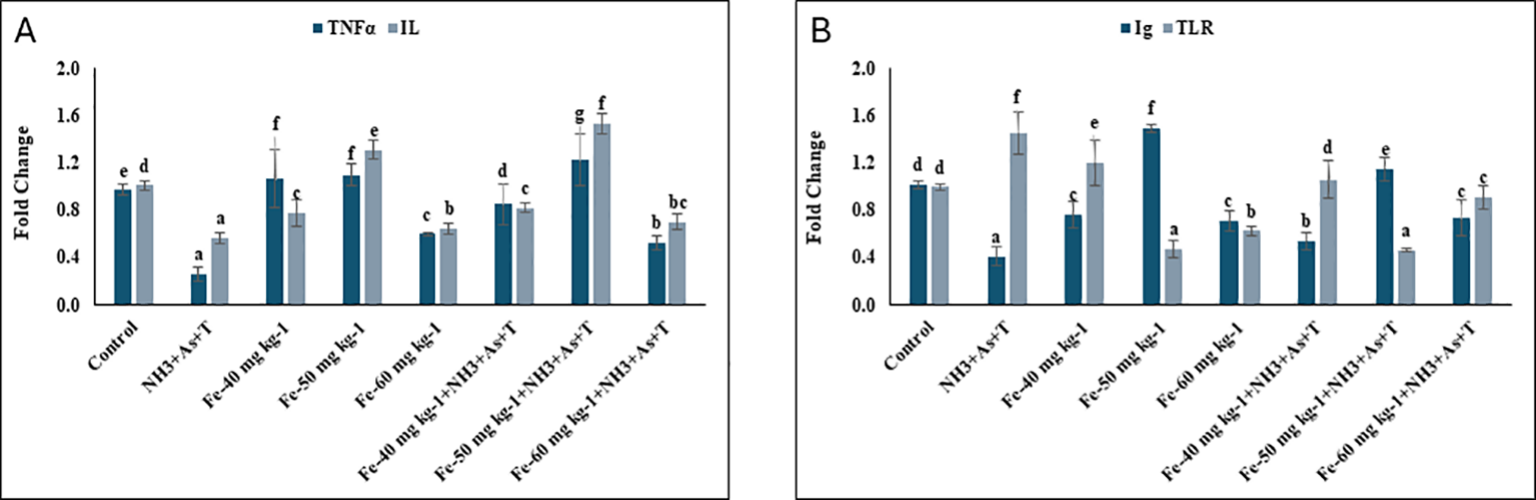
**(A, B)** Effect of dietary iron on cortisol, gene expression of TNFo, IL, Ig and TLR in fish reread under control or multiple stressors. Within endpoints and groups, bars with different superscripts differ significantly (a-g). Data expressed Mean + SE (n=3).

*TNFα* (p=0.0015) and *IL* (p=0.0022) genes were significantly downregulated in fish exposed to ammonia and arsenic toxicity along with high-temperature stress (NH_3_+As+T), compared to the control and Fe fed groups. In contrast, fish fed an Fe at 50 mg kg^-^¹ diet without stressors exhibited significant upregulation of *TNFα* and *IL* expression relative to all other groups (**Figure 3A**). Moreover, immunoglobulin (*Ig*) expression was significantly upregulated (p = 0.0025) in fish fed Fe at 50 mg kg^-^¹ diet without stressors, followed by those fed with same Fe diet under stress conditions, compared to the control and other groups. On the other hand, *TLR* gene expression was notably downregulated (p=0.0018) in groups fed with Fe at 50 and 60 mg kg^-^¹, regardless of stressor exposure, when compared to the control and other treatments. In contrast, fish exposed to stressors (NH_3_+As+T) showed significant downregulation of *Ig* expression and upregulation of *TLR* expression compared to both the control and Fe supplemented groups (**Figure 3B**).

### 3.4 NBT, total protein, albumin, globulin, A:G ratio, blood glucose and MPO affected by concurrent exposure to low dose of ammonia, arsenic and high temperature stress and iron diet protect it

The results related to immunological parameters were particularly noteworthy. NBT (p=0.012), total protein (p=0.002), albumin (0.025), globulin (p=0.04), and myeloperoxidase (MPO) (p=0.0017) levels were significantly reduced in fish exposed to combined stressors (NH_3_+As+T) compared to the control and other treatment groups. However, these immunological attributes were markedly enhanced in fish fed with Fe diet at 50 mg kg^-^¹, both with and without stressor exposure, surpassing even the control group in most cases. In contrast, the albumin-to-globulin (A:G) ratio (p=0.023) exhibited an inverse trend, increasing in the stressor-exposed group and decreasing in the Fe fed at 50 mg kg^-^¹, indicating a shift in immune homeostasis. Additionally, blood glucose levels were significantly elevated (p=0.0016) under combined stress conditions. This stress-induced hyperglycemia was notably ameliorated by dietary Fe at 50 mg kg^-^¹, with and without stressors (**Table 4**).

**Table 4:** Effect of dietary iron on NBT, total protein, albumin, globulin, A:G ratio, blood glucose, and MPO in fish reread under control or multiple stressors.

Exposure	Non-stressors	Stressors (NH_3_+As+T)	Non-stressors			Stressors (NH_3_+As+T)		
Diets (mg kg^-1^)	Control	Control	Fe-40 mg kg^-1^	Fe-50 mg kg^-1^	Fe-60 mg kg^-1^	Fe-40 mg kg^-1^	Fe-50 mg kg^-1^	Fe-60 mg kg^-1^
NBT	0.52 ± 0.01^b^	0.32 ± 0.01^a^	0.53 ± 0.02^b^	0.74 ± 0.04^c^	0.54 ± 0.01^b^	0.57 ± 0.02^b^	0.76 ± 0.03^c^	0.55 ± 0.02^b^
Total protein	1.08 ± 0.01^b^	0.50 ± 0.03^a^	1.09 ± 0.01^b^	1.47 ± 0.12^c^	1.07 ± 0.05^b^	1.12 ± 0.011^b^	1.40 ± 0.04^c^	1.02 ± 0.02^b^
Albumin	0.24 ± 0.01^d^	0.15 ± 0.01^b^	0.10 ± 0.01^a^	0.17 ± 0.02^c^	0.13 ± 0.01^ab^	0.12 ± 0.01^ab^	0.21 ± 0.01^c^	0.16 ± 0.01^b^
Globulin	0.84 ± 0.02^b^	0.35 ± 0.01^a^	0.99 ± 0.04^c^	1.30 ± 0.11^d^	0.95 ± 0.03^c^	1.00 ± 0.06^c^	1.19 ± 0.013^d^	0.86 ± 0.02^b^
A:G ratio	0.28 ± 0.01^c^	0.42 ± 0.02^d^	0.10 ± 0.01^a^	0.13 ± 0.01^ab^	0.14 ± 0.01^ab^	0.12 ± 0.0^ab^	0.18 ± 0.01^b^	0.19 ± 0.01^b^
BG	90.89 ± 0.36^c^	130.14 ± 1.81^d^	80.41 ± 0.61^b^	68.96 ± 0.30^a^	91.12 ± 1.35^c^	92.47 ± 0.65^c^	70.12 ± 1.31^a^	91.76 ± 1.15^c^
MPO	0.34 ± 0.02^b^	0.21 ± 0.02^a^	0.36 ± 0.02^b^	0.47 ± 0.03^d^	0.35 ± 0.01^b^	0.37 ± 0.01^b^	0.47 ± 0.03^d^	0.41 ± 0.02^c^

Values in the same row with different superscript (a, b, c, d, e) differ significantly. NBT, Nitro blue tetrazolium; Total protein, albumin, globulin, g dL^-^1; MPO, Myeloperoxidase; Blood glucose, mgdL^-1^; Data expressed as Mean ± SE (n=3).

### 3.5 *CAT, SOD, GPx*, AChE and *GH* affected by concurrent exposure to low dose of ammonia, arsenic and high temperature stress and iron diet protect it

**Figure 4:**
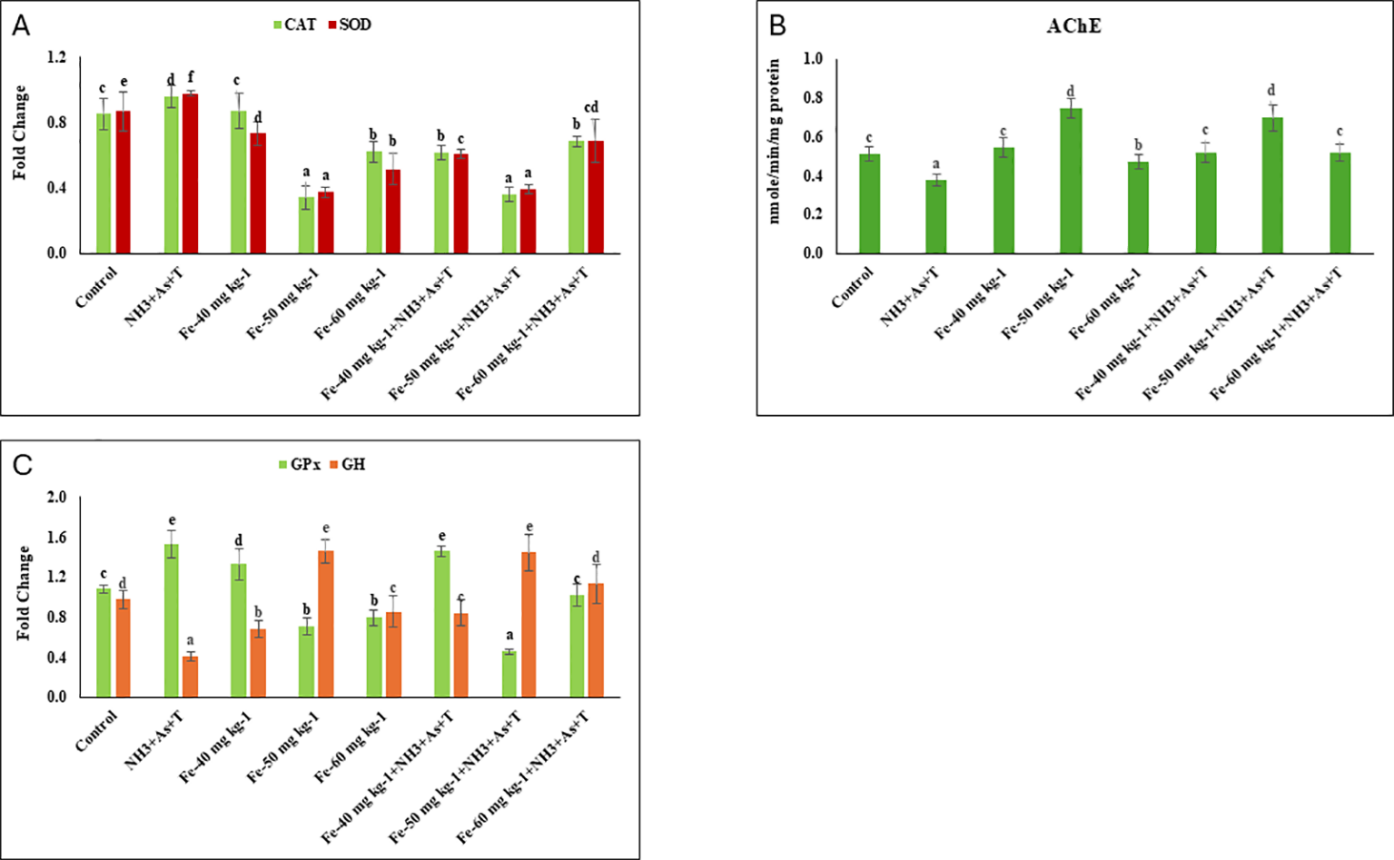
**(A–C)** Effect of dietary iron on cortisol, gene expression of CAT, SOD, 2.0 AChE, GPx, and GH in fish reread under control or multiple stressors. Within endpoints and groups, bars with different superscripts differ significantly (a-f). Data expressed as Mean + SE (n=3).

The expression of catalase (*CAT*) (p=0.0001) and superoxide dismutase (*SOD*) (p=0.012) genes in liver tissue was significantly downregulated in fish fed with Fe at 50 mg kg^-^¹, both with and without stressors, as well as in the 60 mg kg^-^¹ fed groups, compared to the control and other treatments. In contrast, the group exposed to combined stressors (NH_3_+As+T) exhibited significant upregulation of *CAT* and *SOD* expression relative to the control and Fe fed groups (**Figure 4A**). The enzymatic activity of acetylcholinesterase (AChE) was significantly inhibited (p=0.0024) in the stressor group (NH_3_+As+T). However, dietary Fe at 50 mg kg^-^¹, followed by 60 mg kg^-^¹, with or without stressors, significantly enhanced AChE activity compared to the control and other groups (**Figure 4B**). Similarly, glutathione peroxidase (*GPx*) gene expression was markedly downregulated (p=0.0016) with Fe at 50 mg kg^-^¹ diet group, irrespective of stressor exposure, compared to the control and other treatments. Conversely, *GPx* expression was significantly upregulated in the stressor-only group compared to the control and Fe supplemented groups (**Figure 4C**). Additionally, expression of the growth hormone (*GH*) gene was significantly downregulated (p=0.014) in stressor group (NH_3_+As+T). In contrast, *GH* expression was significantly upregulated in fish fed with Fe at 50 mg kg^-^¹, with or without stressors (**Figure 4C**).

### 3.6 *MYST, SMT, GHR1, GHRβ, IGF1X* and *IGF2X* affected by concurrent exposure to low dose of ammonia, arsenic and high temperature stress and iron diet protect it

**Figure 5:**
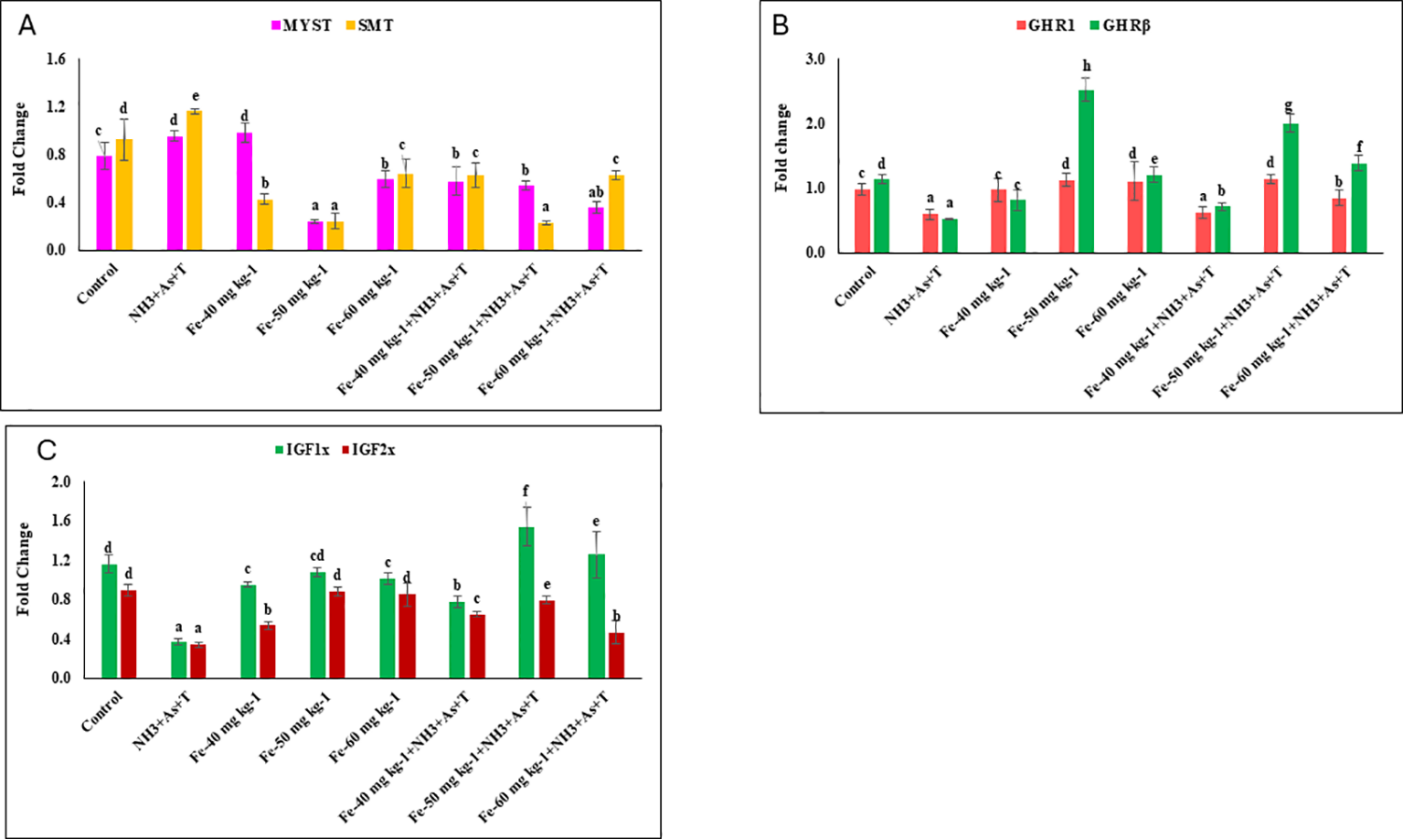
**(A–C)** Effect of dietary iron on cortisol, gene expression of MYST, 1.6 SMT, GHR1, GHRß, IGFIX, and IGF2X in fish reread under control or multipled stressors. Within endpoints and groups, bars with different superscripts differ significantly (a-f). Data expressed as Mean + SE (n=3).

The expression of *MYST* (p=0.001) and *SMT* (p=0.0018) genes were significantly downregulated in fish fed with Fe diets at 40, 50, and 60 mg kg^-^¹, with and without stressors, compared to the control and other groups. In contrast, significant upregulation of *MYST* and *SMT* expression was observed in the group exposed to stressors group (NH_3_+As+T) (**Figure 5A**). Furthermore, *GHR1* (p=0.025) and *GHRβ* (p=0.001) gene expressions were markedly upregulated in fish fed with Fe at 50 mg kg^-^¹, followed by 60 mg kg^-^¹, with or without stressors, compared to control and other groups. Conversely, stressor group (NH_3_+As+T) exhibited significant downregulation of GHR1 and GHRβ (**Figure 5B**). Additionally, the expression of *IGF1X* (p = 0.018) and *IGF2X* (p = 0.012) was significantly downregulated in fish exposed to ammonia, arsenic, and high-temperature stress, compared to the control and Fe fed groups. However, Fe at 50 mg kg^-^¹ and concurrently exposed to stressors showed significant upregulation of *IGF1X* and *IGF2X.* Notably, the expression of these genes in Fe-fed groups (40, 50, and 60 mg kg^-^¹) without stressors remained comparable to the control group (**Figure 5C**).3.7. Growth performance indicators affected by concurrent exposure to low dose of ammonia, arsenic and high temperature stress and Iron diet protect it.

Final weight gain percentage was significantly higher (p=0.0022) in fish fed with Fe diet at 50 mg kg^-^¹, both with and without stressors, followed Fe diet at 60 mg kg^-^¹, compared to the control and other groups. Notably, growth in the 50 mg kg^-^¹ Fe group was approximately 60% greater than that control group. Additionally, the specific growth rate (SGR) was significantly elevated (p=0.0014) in fish fed with Fe at 50 and 60 mg kg^-^¹ diets without stressors, and in the group fed with Fe at 50 mg kg^-^¹ with stressors, compared to the control and other treatments. Similarly, both the protein efficiency ratio (PER) (p=0.013) and daily growth index (DGI) (p=0.016) were significantly higher in the groups fed with Fe at 50 and 60 mg kg^-^¹, irrespective of stressor exposure, compared to the control and other groups. Relative feed intake (RFI) was also significantly enhanced (p=0.0018) with Fe at 40, 50, and 60 mg kg^-^¹ diet, with and without stressors. In contrast, the feed conversion ratio (FCR) (p=0.012) showed an inverse trend to other growth metrics, being significantly reduced in the Fe at 50 mg kg^-^¹ diet with and without stressors (NH_3_+As+T). Overall, all growth performance parameters were significantly affected by concurrent exposure to low doses of ammonia, arsenic, and high-temperature stress (**Table 5**).

**Table 5:** Effect of dietary iron on growth performance viz. final body weight gain (%), FCR, SGR, PER, DGI (%) and RFI in fish reread under control or multiple stressors.

Exposure	Non-stressors	Stressors (NH_3_+As+T)	Non-stressors			Stressors (NH_3_+As+T)		
Diets (mg kg^-1^)	Control	Control	Fe-40 mg kg^-1^	Fe-50 mg kg^-1^	Fe-60 mg kg^-1^	Fe-40 mg kg^-1^	Fe-50 mg kg^-1^	Fe-60 mg kg^-1^
Final weight gain %	86.77 ± 2.90^b^	40.74 ± 2.39^a^	90.93 ± 2.69^bc^	139.0 ± 1.69^g^	125.52 ± 2.52^e^	92.35 ± 5.10^c^	130.25 ± 1.55^f^	116.8 ± 2.77^d^
FCR	3.24 ± 0.10^b^	5.88 ± 0.30^c^	3.16 ± 0.06^b^	2.37 ± 0.01^a^	2.31 ± 0.04^a^	3.14 ± 0.11^b^	2.39 ± 0.03^a^	2.59 ± 0.04^a^
SGR	0.69 ± 0.02^b^	0.38 ± 0.02^a^	0.72 ± 0.01^b^	0.97 ± 0.02^e^	0.90 ± 0.02^d^	0.73 ± 0.03^b^	0.93 ± 0.01^e^	0.86 ± 0.02^b^
PER	0.89 ± 0.01^b^	0.54 ± 0.04^a^	0.98 ± 0.02^c^	1.31 ± 0.02^ef^	1.40 ± 0.03^f^	0.92 ± 0.06^bc^	1.23 ± 0.02^e^	1.14 ± 0.03^d^
DGI (%)	0.99 ± 0.01^b^	0.53 ± 0.02^a^	1.06 ± 0.03^b^	1.40 ± 0.02^d^	1.37 ± 0.02^d^	1.03 ± 0.01^b^	1.42 ± 0.03^d^	1.30 ± 0.02^c^
RFI	280.92 ± 1.46^b^	238.21 ± 3.05^a^	286.79 ± 3.65^c^	329.98 ± 5.30^f^	289.87 ± 2.0^c^	288.78 ± 5.58^c^	311.06 ± 0.80^e^	301.82 ± 3.25^d^

Values in the same row with different superscript (a, b, c, d, e) differ significantly. Data expressed as Mean ± SE (n=3). FCR, feed conversion ratio; SGR, specific growth rate; PER, protein efficiency ratio; DGI, Daily growth index; TGC, Thermal growth coefficient; RFI, relative feed intake.

### 3.8 ALT, AST, LDH and MDH affected by concurrent exposure to low dose of ammonia, arsenic and high temperature stress and Iron diet protect it

Activities of ALT (p=0.015), AST (p=0.0012), LDH (p=0.001), and MDH (p=0.0017) in liver and gill tissues were significantly elevated in fish exposed to concurrent low doses of ammonia, arsenic, and high-temperature stress, compared to the control and Fe diet groups. In contrast, ALT activity in liver tissue was significantly reduced in fish fed Fe at 50 mg kg^-^¹, with and without stressors, followed by those fed with Fe at 40 mg kg^-^¹, compared to the control and other groups. Similarly, ALT activity in gill tissue and AST activity in liver tissue were significantly reduced in the group fed Fe at 50 mg kg^-^¹ without stressors, compared to all other treatments. AST activity in gill tissue was significantly lower in the group fed Fe at 60 mg kg^-^¹, followed by the 50 mg kg^-^¹ without stressors, compared to all other groups. LDH activity was notably reduced in liver tissue with Fe at 50 mg kg^-^¹, followed by 60 mg kg^-^¹. In gill tissue, LDH activity was lowest in the group fed with Fe at 50 mg kg^-^¹ without stressors, followed by Fe at 40, 50, and 60 mg kg^-^¹ with stressors. Additionally, MDH activity in both liver and gill tissues were significantly reduced with Fe supplementation at 50 mg kg^-^¹, followed by 40 mg kg^-^¹ without stressors, in comparison to the control and other treatment groups (**Table 6**).

**Table 6:** Effect of dietary iron on alanine amino transferase (ALT), aspartate amino transferase (AST), lactate dehydrogenase (LDH) and malate dehydrogenase (MDH) in fish reread under control or multiple stressors. .

Exposure	Non-stressors	Stressors (NH_3_+As+T)	Non-stressors			Stressors (NH_3_+As+T)		
Diets (mg kg^-1^)	Control	Control	Fe-40 mg kg^-1^	Fe-50 mg kg^-1^	Fe-60 mg kg^-1^	Fe-40 mg kg^-1^	Fe-50 mg kg^-1^	Fe-60 mg kg^-1^
ALT-L	3.64 ± 0.37^c^	9.96 ± 0.56^f^	3.07 ± 0.23^b^	1.88 ± 0.23^a^	4.04 ± 0.34^d^	4.92 ± 0.75^e^	1.94 ± 0.34^a^	4.70 ± 0.55^e^
ALT-G	3.14 ± 0.36^b^	7.47 ± 0.75^f^	4.22 ± 0.33^d^	1.81 ± 0.30^a^	4.75 ± 0.55^de^	3.69 ± 0.59^c^	5.60 ± 0.50^e^	5.71 ± 0.37^e^
AST-L	2.98 ± 0.37^b^	6.96 ± 0.81^e^	5.23 ± 0.39^c^	1.59 ± 0.10^a^	2.99 ± 0.45^b^	6.32 ± 0.89^d^	5.11 ± 0.67^c^	6.79 ± 0.51^e^
AST-G	5.79 ± 0.42^c^	10.37 ± 0.53^e^	6.76 ± 0.80^d^	3.90 ± 0.36^b^	2.65 ± 0.35^a^	5.45 ± 0.81^c^	6.86 ± 0.68^d^	5.53 ± 0.80^c^
LDH-L	2.24 ± 0.30^c^	4.37 ± 0.22^d^	2.29 ± 0.15^c^	1.15 ± 0.08^a^	1.96 ± 0.19^b^	2.20 ± 0.17^c^	2.14 ± 0.08^c^	2.29 ± 0.34^c^
LDH-G	3.61 ± 0.18^c^	7.11 ± 0.42^d^	3.56 ± 0.27^c^	1.99 ± 0.13^a^	3.56 ± 0.20^c^	2.91 ± 0.19^b^	2.88 ± 0.49^b^	2.66 ± 0.43^b^
MDH-L	1.34 ± 0.05^c^	2.97 ± 0.19^d^	1.09 ± 0.19^b^	0.78 ± 0.07^a^	1.32 ± 0.08^c^	1.35 ± 0.06^c^	1.33 ± 0.02^c^	1.37 ± 0.03^c^
MDH-G	2.46 ± 0.12^c^	4.12 ± 0.24^d^	1.57 ± 0.23^b^	0.84 ± 0.15^a^	1.79 ± 0.14^bc^	1.84 ± 0.09^bc^	1.81 ± 0.07^bc^	1.88 ± 0.05^bc^

Values in the same row with different superscript (a, b, c, d, e) differ significantly. Data expressed as Mean ± SE (n=6). Alanine amino transferase (ALT): Nanomole of sodium pyruvate formed/mg protein at 37 °C; aspartate amino transferase (AST): Nanomole od oxaloacetate released/min/mg protein at 37 °C; Lactate dehydrogenase (LDH) and malate dehydrogenase (MDH): Units/mg protein.

### 3.9 Iron diet reduced the arsenic bioaccumulation in different fish tissues

Arsenic concentrations in the experimental water varied markedly across treatment groups. The control group showed As concentration of 0.02 µg L^-^¹, while the group exposed to combined stressors (ammonia, arsenic, and high temperature) showed a substantially elevated concentration of 2396 µg L^-^¹. Moreover, Fe fed groups at 40, 50, and 60 mg kg^-^¹ diets without stressors, As levels were below the detection limit (BDL). However, in Fe fed groups with stressors, As concentrations were reduced to 565 µg L^-^¹ (40 mg kg^-^¹), 365 µg L^-^¹ (50 mg kg^-^¹), and 612 µg L^-^¹ (60 mg kg^-^¹) (**Table 6**). Furthermore, tissue-specific arsenic bioaccumulation was assessed. The highest accumulation was determined in the kidney (9.39 mg kg^-^¹), followed by the liver (8.11 mg kg^-^¹), gill (5.06 mg kg^-^¹), muscle (1.37 mg kg^-^¹), and brain (0.76 mg kg^-^¹) in the stressors group (NH_3_+As+T). Notably, the group fed with Fe at 50 mg kg^-^¹ with stressors showed the lowest As accumulation in muscle tissue (0.01 mg kg^-^¹), indicating a protective effect of Fe supplementation (**Table 7**).

**Table 7:** Effect of dietary iron on experimental water and detoxification of arsenic in different fish tissues reared under control and multiple stress condition.

Exposure/Diets (mg kg^-1^)	Water (µg L^-1^)	Liver (mg kg^-1^)	Kidney (mg kg^-1^)	Gill (mg kg^-1^)	Muscle (mg kg^-1^)	Brain (mg kg^-1^)
Ctr/Ctr	0.02 ± 0.00	BDL	BDL	BDL	BDL	BDL
NH_3_+As+T/Ctr	2396.18 ± 21.27	8.11 ± 0.18	9.39 ± 0.038	5.06 ± 0.17	1.37 ± 0.02	0.76 ± 0.02
Fe-40 mg kg^-1^	BDL	BDL	BDL	BDL	BDL	BDL
Fe-50 mg kg^-1^	BDL	BDL	BDL	BDL	BDL	BDL
Fe-60 mg kg^-1^	BDL	BDL	BDL	BDL	BDL	BDL
NH_3_+As+T-Fe-40	565.57 ± 11.18	2.03 ± 0.02	2.84 ± 0.07	1.11 ± 0.02	0.20 ± 0.01	0.14 ± 0.0
NH_3_+As+T-Fe-50	365.28 ± 6.29	0.81 ± 0.01	0.47 ± 0.02	0.15 ± 0.01	0.01 ± 0.0	0.11 ± 0.0
NH_3_+As+T-Fe-60	612.49 ± 15.25	2.05 ± 0.04	1.63 ± 0.012	1.10 ± 0.04	0.57 ± 0.01	0.23 ± 0.01

Data expressed as Mean ± SE (n=3). BDL: Below detection limit.

### 3.10 Iron diet protect fish against pathogenic infection

**Figure 6:**
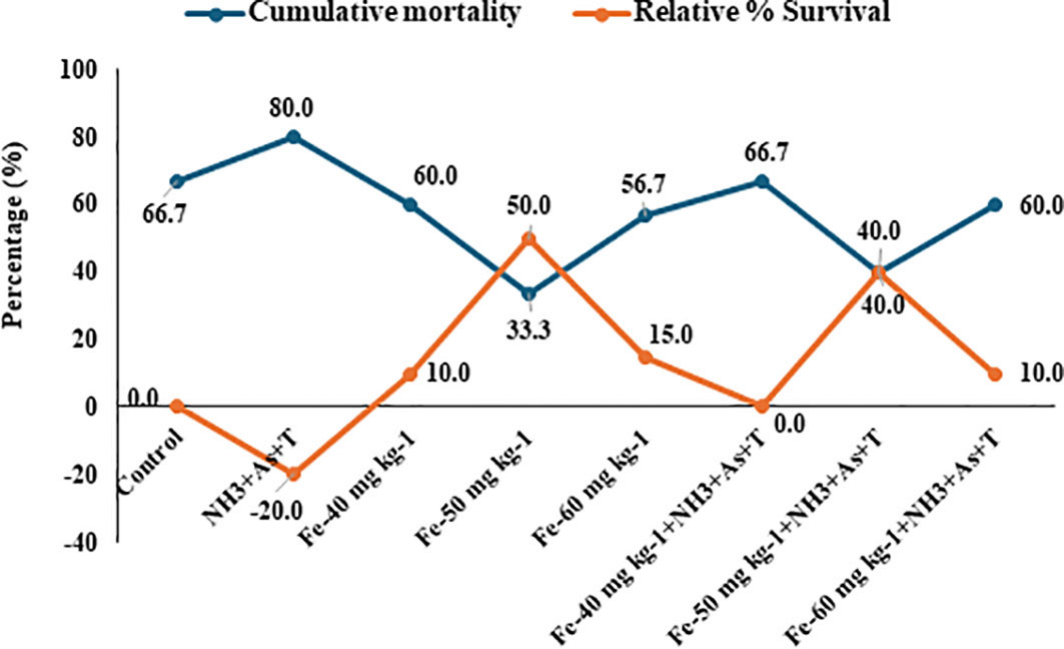
Effect of dietary iron on cumulative mortality and relative percentage survival (%) in fish reared under control or multiple stressors.

In the present study, fish were challenged with *Aeromonas hydrophila* at the end of the experimental period, and mortality was monitored for one week and data are presented in **Figure 6**. Cumulative mortality were significantly among the treatment groups: 66% in the control group, 80% in the stressor-exposed group (NH_3_+As+T), and 60%, 33%, 56%, 66%, 40%, and 60% in the Fe supplemented groups at 40, 50, and 60 mg kg^-^¹ diet, with or without stressors, respectively. Correspondingly, the relative percentage survival (RPS) was lowest in the stressor group (-20%), while Fe supplementation improved survival of the fish. RPS values were 10%, 50% and 15% with Fe at 40 mg kg^-^¹, 50 mg kg^-^¹, and 60 mg kg^-^¹ without stressors respectively. The group fed with Fe at 40 mg kg^-^¹, 50 mg kg^-^¹, and 60 mg, the RPS was 0% 40% and 10% respectively. These results highlight the enhanced disease resistance provided by dietary Fe, particularly at 50 mg kg^-^¹, even under stress conditions.

## 4 Discussion

The present investigation addresses timely and relevant issues concerning pollution and climate change in aquatic ecosystems. Abiotic stressors significantly affect the development and survival of aquatic organisms, including fish, ultimately reducing their productivity (47). This study explores the molecular mechanisms underlying fish responses to key abiotic stressors ammonia and arsenic toxicity, and elevated temperature focusing particularly on gene regulation. Stress responses in fish are categorized into primary, secondary, and tertiary response.

Cortisol, a principal hormone involved in the primary stress response, is regulated by the hypothalamus-pituitary-interrenal (HPI) axis. Dietary iron (Fe) supplementation at 40 and 50 mg kg^-^¹ significantly reduced cortisol, likely by modulating HPI axis activity, which governs cortisol biosynthesis and secretion in the interrenal tissue (48, 49). Studies conducted by Javed and Usmani (50) and Pettem et al. (51) found that metal toxicity disrupts metabolic activities, elevates cortisol levels, and impairs energy homeostasis. Although, A potential limitation of the present study is the use of a single reference gene (β-actin) for normalization of qPCR data. Although *β-actin* has been widely used in previous studies under related stress conditions, the use of multiple reference genes provides more robust normalization and reduces potential bias in relative expression estimates. Heat shock proteins (HSPs) function as molecular chaperones that facilitate the proper folding of nascent and stress denatured proteins, inhibit protein aggregation, and protect polypeptides from proteasomal degradation (52). In the present study, dietary Fe at 50 mg kg^-^¹ led to a downregulation of HSP gene expression. In contrast, concurrent exposure to NH_3_+As+T induced a marked upregulation of liver *HSP70* expression, likely through the activation of stress responsive transcription factors that interact with heat shock elements (53, 54). Additionally, the expression of *DDIP*, a gene implicated in cellular protection against DNA damage, was reduced following Fe at 50 mg kg^-^¹, possibly reflecting enhanced efficiency of DNA repair mechanisms mediated by iron dependent enzymes. Iron supplementation further ameliorated oxidative stress, as indicated by decreased reactive oxygen species (ROS) production, elevated cytochrome *P450 (CYP450*) expression, and improved coordination between oxidative phosphorylation and overall energy metabolism (55).

Furthermore, cytochrome P450 (*CYP450*) gene expression was markedly altered under concurrent exposure to ammonia, arsenic, and elevated temperature stress. In contrast, dietary Fe supplementation at 50 mg kg^-^¹ effectively restored and stabilized *CYP450* expression. This modulation can be attributed to the indispensable role of Fe in heme biosynthesis and its regulatory involvement in key enzymes, including ferrochelatase (56)) and δ-aminolevulinic acid synthetase (57). The Fe enriched diet also enhanced NADPH availability and stimulated *CYP450* reductase (P450-RED) activity by upregulating critical enzymes of the pentose phosphate pathway, namely glucose-6-phosphate dehydrogenase (*G6PDH*) and 6-phosphogluconate dehydrogenase (*6PGDH*). Notably, dietary Fe at 50 mg kg^-^¹ resulted in the downregulation of inducible nitric oxide synthase (*iNOS*) gene expression, in contrast to the pronounced induction observed under combined stress conditions (NH_3_+As+T). This suppressive effect of Fe on *iNOS* expression may be associated with its capacity to limit reactive oxygen species (ROS) accumulation through nitric oxide synthase (NOS) dependent regulatory mechanisms (58). Additionally, Fe supplementation appeared to promote cellular hypertrophy and induce the expression of cytoprotective and anti-apoptotic genes, including Bcl-2, survivin, leukemia inhibitory factor (LIF), as well as hypertrophic mediators such as endothelin-1 (ET 1), thereby reducing H_2_O_2_-induced necrotic damage (59). Ammonia exposure exerted a particularly strong influence on *iNOS* expression, likely due to its hepatotoxic effects following systemic distribution. Although iNOS is commonly associated with oxidative stress, its induction may also contribute to cellular defense mechanisms against oxidative injury (60).

*Caspase 3a* and *3b* are pivotal apoptotic effector genes that mediate the execution phase of programmed cell death. In the present investigation, dietary Fe supplementation at 50 mg kg^-^¹ significantly suppressed the expression of *CAS 3a* and *3b*, potentially reflecting Fe mediated regulation of protein disulfide isomerase (PDI), an enzyme implicated in apoptosis through its influence on caspase activation (61). In addition, iron contributes to the stabilization of redox sensitive thiol groups and facilitates the coordination of trace metals such as zinc and copper at enzymatic active sites (62). Notably, metallothionein (*MT*) gene expression was also reduced in fish receiving the Fe supplemented diet. Although Fe is essential for erythropoiesis and reticulocyte maturation (63), *MT* expression is predominantly upregulated in response to heavy metal exposure and environmental stressors, including ammonia, arsenic, and elevated temperature (64, 65). This stress induced *MT* activation is regulated by metal-responsive transcription factor 1 (MTF-1), which undergoes phosphorylation under adverse conditions (66). Furthermore, combined abiotic stress (NH_3_+As+T) markedly elevates cellular energy requirements, particularly for the maintenance of ionic gradients and cellular homeostasis, processes that rely heavily on ATPase activity. Dietary Fe supplementation supports cellular energy metabolism by enhancing ATP generation, thereby preserving cellular integrity and physiological function under stressful conditions (16).

In the present study, the transcriptional levels of tumor necrosis factor-α (*TNF-α*), interleukins *(ILs*), and immunoglobulin *(Ig*) genes were markedly suppressed under combined abiotic stress conditions (NH_3_+As+T), whereas their expression was significantly elevated in fish receiving an Fe supplemented diet. In contrast, toll-like receptor (*TLR*) gene expression was induced by stress exposure but attenuated following Fe supplementation. These genes constitute key components of cytokine-mediated immune signaling pathways, and dietary Fe appears to modulate immune function, particularly through regulation of TNF-α synthesis (67). The Fe enriched diet promoted *TNF-α* production, a cytokine essential for reinforcing immune competence under both basal and stress challenged conditions (64, 65). This enhanced TNF-α expression may be associated with increased ferritin synthesis and activation of β-cell-activating factor (BAFF), processes linked to nuclear factor-κB (NF-κB) signaling (68). Additionally, *IL* gene expression was substantially upregulated in Fe fed fish. Although the underlying mechanisms remain to be fully elucidated, this response may involve stimulation of CD4^+^ T cell proliferation and induction of hepcidin synthesis (69, 70). Conversely, the downregulation of *TLR* expression in Fe supplemented groups may reflect iron and heme sequestration during inflammatory or infectious processes, thereby fine tuning immune signaling under stress conditions (71). As TLRs play a central role in initiating innate immune responses via NF-κB activation, their stress-induced upregulation likely represents a compensatory response to immunosuppression. Notably, Fe supplementation also enhanced *Ig* gene expression, potentially through increased T cell activation and elevated immunoglobulin synthesis, thereby strengthening adaptive immune responses (72). To further evaluate immune status, several hematological and biochemical markers including nitro blue tetrazolium (NBT) activity, total protein, albumin, globulin, the albumin-to-globulin (A:G) ratio, myeloperoxidase (MPO) activity, and blood glucose levels were assessed. Dietary Fe supplementation significantly improved all these parameters under both control and stress conditions. Elevated NBT activity in Fe fed fish indicated enhanced phagocytic capacity and immune competence, whereas exposure to combined stressors (NH_3_+As+T) resulted in reduced NBT activity, indicative of immunosuppression (73). Similarly, MPO activity, which mediates hypochlorous acid production during the neutrophil respiratory burst, was increased with Fe supplementation, further supporting improved innate immune function. Blood glucose levels, a sensitive indicator of physiological stress, were significantly elevated under stress exposure but normalized following Fe supplementation. This effect may be linked to the role of Fe in regulating glucose metabolism and maintaining adequate blood perfusion to vital organs (16). Moreover, iron plays an essential role in the transport of drugs, vitamins, hormones, and bilirubin, as well as in lipid metabolism, underscoring its multifaceted contribution to overall fish health and stress resilience (74).

In the present investigation, liver expression of key antioxidant enzymes CAT, SOD and GPx was significantly elevated under combined abiotic stress conditions (NH_3_+As+T), reflecting an enhanced oxidative stress. In contrast, the transcriptional levels of these antioxidant genes were reduced in fish receiving Fe supplemented diets, indicating a mitigating effect of Fe on oxidative stress. This protective response may be attributed to the ability of dietary Fe to reduce reactive oxygen species (ROS) accumulation and to support oxygen transport and redox regulatory proteins that maintain cellular redox balance in fish (75). Iron is integral to numerous physiological processes, including the functional activity of antioxidant enzymes such as GPx and SOD, which safeguard cells against oxidative injury by restricting free radical generation and lipid peroxidation (76). Catalase, a heme dependent enzyme, in conjunction with SOD, plays a central role in the detoxification of hydrogen peroxide and the regulation of intracellular ROS, thereby enhancing overall antioxidant defense capacity (76). Additionally, acetylcholinesterase (AChE) activity, a critical determinant of normal neural function, was markedly suppressed following simultaneous exposure to ammonia, arsenic, and elevated temperature. Conversely, dietary Fe supplementation significantly restored AChE activity, likely through iron mediated modulation of signaling pathways involved in neuroprotection and synaptic function (16).

Growth performance parameters, including final weight gain (%), FCR, SGR, PER, DGI %, and RFI, were significantly improved in fish receiving Fe supplemented diets under both control and combined abiotic stress conditions (NH_3_+As+T). Iron acts as an effective growth promoting micronutrient by maintaining systemic iron homeostasis, supporting metabolic efficiency, and enhancing nutrient digestion, absorption, and feed utilization (77). Consequently, dietary Fe supplementation plays a crucial role in optimizing growth performance in fish. At the molecular level, Fe supplementation modulated the expression of multiple growth-regulatory genes, including growth hormone (*GH*), somatostatin (*SMT*), myostatin (*MYST*), growth hormone receptors (*GHRβ* and *GHR1*), and insulin-like growth factors (*IGF1X* and *IGF2X*). Fe enriched diets upregulated the majority of these genes, thereby contributing to the observed improvements in growth indices. Notably, *SMT* and *MYST* expression was significantly downregulated by Fe at 50 mg kg^-^¹, which may be linked to the inhibitory role of myostatin in myoblast proliferation and its involvement in muscle fiber hypertrophy and terminal differentiation (78). Insulin-like growth factors, particularly *IGF1X1* and *IGF1X2*, are central regulators of protein, carbohydrate, lipid, and mineral metabolism and exert a direct influence on somatic growth (79). Growth hormone stimulates liver *IGF* synthesis through receptor-mediated signaling, thereby amplifying growth responses. In addition, iron has been shown to upregulated *IGF* secretion and signaling, including via central nervous system pathways, further reinforcing its regulatory role in growth modulation (80). To evaluate liver health under stress exposure, key biochemical indicators such as ALT, AST, LDH and MDH were assessed. Combined exposure to ammonia, arsenic, and elevated temperature resulted in significant elevations in these enzymes, indicative of liver stress or tissue injury. In contrast, dietary Fe supplementation markedly reduced their activities, reflecting improved liver function and attenuation of stress induced liver damage.

Arsenic detoxification is largely involved by the regulation of cytochrome P450 (*CYP450*) dependent on metabolic pathways and the capacity of vital organs to eliminate toxicants from the body. In the present study, arsenic accumulation was highest in the kidney and liver, followed by the gills. However, dietary Fe supplementation markedly enhanced arsenic depuration across all analyzed tissues (16, 81), likely through Fe mediated modulation of detoxification associated genes, particularly *CYP450* (16).

In addition to its role in metal detoxification, the Fe enriched diet conferred protection against bacterial challenge, as reflected by improved cumulative mortality rates and higher relative percent survival (RPS) in Fe fed fish. Iron is essential for effective antibacterial defense and phagocytic function and contributes to membrane fluidity as well as the activity of antimicrobial enzymes, including lysozyme (16, 82). Furthermore, Fe interacts with microbial siderophores, such as enterobactin, which are produced by pathogens to acquire iron, thereby restricting microbial proliferation and supporting host immune responses (83). The siderophore Fe complex binds to specific cell surface receptors, triggering the release of ferric reductases that catalyze the reduction of ferric iron (Fe³^+^) to its more soluble ferrous form (Fe²^+^). In addition, host derived lipocalin 2 plays a crucial role in sequestering siderophores, thereby limiting iron availability to invading pathogens and contributing to effective infection control (84). Higher mortality was observed in the control group and was attributed to bacterial infection occurring after unseen handling stress and increased susceptibility due to the absence of any protective intervention, such as the lack of dietary iron supplementation in this group. Which indicated that essentiality of iron diets in fish feed reared under multiple abiotic stress condition.

## 5 Conclusions

The present investigation addresses environmental concerns such as ammonia, and arsenic toxicity as well as high temperature, which significantly disrupt aquatic ecosystems and adversely affect aquatic organisms, particularly fish. Dietary supplementation with Fe has shown promise in mitigating the adverse effects of multiple stressors, including ammonia, arsenic, and high temperature (NH_3_+As+T). Fe containing diets modulate stress-responsive gene expression, thereby enhancing the resilience of fish to these environmental challenges. Specifically, Fe supplementation at 50 mg kg^-^¹ was effective in promoting arsenic detoxification across various fish tissues and in reducing susceptibility to pathogenic infections. Moreover, Fe at 50 mg kg^-1^ diet significantly improved the expression of genes associated with growth performance, antioxidant defense, immune response, and apoptosis regulation. Collectively, these findings highlight the potential of Fe supplementation as a strategic intervention to boost fish health and performance under the compounded stressors of ammonia, arsenic, and high temperature (NH_3_+As+T). Moreover, the present research finding can be implemented for future research for management of different abiotic in aquaculture sector using nano-formulated diet.

## Data Availability

The datasets presented in this study can be found in online repositories. The names of the repository/repositories and accession number(s) can be found in the article/**Supplementary Material**.
