# Supplementary material for: Mitigating combined toxic effects of arsenic, ammonia, and high temperature through dietary Iron in fish
Source: Front Immunol. 2026 Mar 11;17:1733912. doi: 10.3389/fimmu.2026.1733912 (PMC13012918; doi:10.3389/fimmu.2026.1733912)
Supplement: Supplementary file 1 [file Table1.doc]

Table S1: Physico-chemical parameters of water during the experimental period of 40 days

| Treatments | Temperature (°C) | pH | DO (mgL^-1^ ) | Hardness (mgL^-1^ ) | Ammonia-N (mgL^-1^ ) |
| --- | --- | --- | --- | --- | --- |
| Ctr | 24.6-28.8 | 7.4-7.7 | 6.4-7.2 | 234.7-232.3 | 0.18-0.21 |
| As+NH_3_+T | 33.8-34.17 | 7.3-7.6 | 6.2-7.1 | 231.3-233.3 | 0.20-0.21 |
| Fe at 40 mg kg^-1^ diet | 24.78-28.5 | 7.1-7.8 | 6.6-7.2 | 228.3-231.6 | 0.20-0.23 |
| Fe at 50 mg kg^-1^ diet | 24.7-28.4 | 7.3-7.6 | 6.3-7.2 | 228.2-231.4 | 0.19-0.22 |
| Fe at 60 mg kg^-1^ diet | 24.6-28.6 | 7.2-7.5 | 6.4.-6.8 | 229.4-230.45 | 0.18-0.19 |
| Fe at 40 mg kg^-1^ diet+As+NH_3_+T | 34.3-34.6 | 7.4-7.8 | 6.1-6.4 | 230.4-231.85 | 0.19-0.21 |
| Fe at 50 mg kg^-1^ diet+As+NH_3_+T | 34.2-35.1 | 7.6-7.9 | 6.0-6.3 | 231.5-231.7 | 0.18-0.20 |
| Fe at 60 mg kg^-1^ diet+As+NH_3_+T | 35.1-35.4 | 7.5-7.8 | 6.1-6.4 | 230.14-231.5 | 0.19-0.21 |

(mean= n-3)
